# AllpaDB: a compiled repository of harmonized soil legacy data and research data from Peru

**DOI:** 10.1038/s41597-026-07558-9

**Published:** 2026-06-25

**Authors:** Carlos Mestanza, Renzo Huerta, Rigel Llerena, Oscar Panduro, Miguel Culqui, Cristhian Oscco, Melissa Quispe, Lily Tello, Julio Nazario, Alexandre ten Caten, César Beltrán

**Affiliations:** 1 Pontificia Universidad Católica del Perú, Lima, Peru; 2 Universidad Nacional Agraria La Molina, Lima, Peru; 3 Universidade Federal de Santa Catarina, Florianópolis, Brazil

**Keywords:** Agriculture, Geomorphology

## Abstract

Soil data from a country are a key tool for developing strategies to achieve sustainable development goals. Here, we present the AllpaDB dataset, which contains harmonized data compiled from various legacy soil data and research in Peru. The dataset includes both spatial and non-spatial information from soil profiles collected throughout the country. The data were processed to correct inconsistencies, and key variables were harmonized between different laboratory methods to ensure their usability. This dataset is a valuable resource for a wide range of applications, including regional soil mapping, environmental modelling, and land use planning.

## Background & Summary

Soil is a layered natural material at the Earth’s surface, formed through physical, chemical, and biological processes, which supports terrestrial ecosystems and humanity^1^. Soil information is key to conserving its multifunctional role in food security, climate mitigation, and biodiversity conservation, while enabling science-based sustainable management practices to achieve the United Nations Sustainable Development Goals by 2030^2^. An important source of information is soil legacy data, which consists of historical soil data and information, often collected over 70 years and primarily stored in paper formats like soil survey reports and maps; these are at risk of being lost and require rescue efforts for digitization and compilation into usable databases^3^. Many regions globally are actively engaged in recovering legacy data and integrating it into harmonized formats^4^.

National legacy soil data often exhibit discrepancies in profile descriptions, localization standards, laboratory methods, map scales, and database organization^5^. Integrating soil data from diverse sources requires harmonization to address potential discrepancies arising from differences in sampling protocols or analytical methods^6^. Data harmonization means that results are comparable irrespective of the measurement procedure used and where or when a measurement was made^7^, although in practice, full independence is seldom achievable as methodological differences can often be corrected only approximately. Soil data harmonization involves the process of combining and standardizing heterogeneous datasets to enable their integration into unified database systems for purposes like digital soil mapping^8^. Harmonization processes can be applied for local data integration^9^ or for global-scale applications^10^. In Latin America, efforts to publish legacy soil databases are limited, and harmonization initiatives are even scarcer, with most efforts restricted to local applications, as seen in Argentina^11^, Chile^12^, Brasil^13^, Colombia^14^, and Ecuador^15^.

Local soil data for Peru is severely limited in both quantity and accessibility. International databases such as World Soil Information Service – WoSIS^16^ and Latin America and Caribbean Soil Information System – SISLAC^17^ report 238 and 148 georeferenced soil data respectively, though these figures do not account for overlapping samples between them. This scarcity of soil information constrains research and decision-making for sustainable land management of Peruvian ecosystems. Historical records indicate that the earliest soil studies in Peru date back to 1920 and soil surveys aligned with USDA standards did not emerge until 1945^18^. Currently, soil surveys continue to be conducted, regulated by Supreme Decree N° 013-2010-AG^19^. This legacy soil data resides in technical documents; its current format prevents effective processing and analysis.

Addressing this challenge, we collected soil legacy data from government documents and scientific investigations, including both georeferenced and non-georeferenced soil information. Data harmonization processes were applied to standardize laboratory results, but temporal differences between samples were not harmonized. AllpaDB dataset offers the capability for continuous updates by integrating recently published data. The dataset supports digital soil mapping, land-use capacity assessments, and hypothesis-driven research on soil properties in Peru. The name ‘allpaDB’ is derived from the Quechua word allpa, meaning ‘soil’, reflecting the origin and focus of the dataset on Peruvian soils. This dataset is made available for non-commercial purposes under the Creative Commons Attribution 4.0 International License (CC BY 4.0). Users employing this database in their publications are requested to provide proper attribution by citing both the dataset and this publication.

## Methods

This section is divided into three subsections: data collection, soil analysis, and data processing. The data collection subsection describes the information sources used to generate the dataset. Soil analysis details the different laboratory methods reported in the documents for the soil samples. Finally, Data processing outlines the procedure followed to convert the raw data into corrected and harmonized data.

### Data collection

The soil data were collected from three primary sources: historical documents (HD) from National Office for Natural Resource Evaluation (ONERN), National Institute of Natural Resources (INRENA), and Ministry of Agriculture (MINAG); governmental reports from Ecological Economic Zoning (ZEE); and research studies of universities and research institutes (RES).

The HD included both virtual and physical files. A total of 133 HD documents were identified, with 103 containing valid soil data. The virtual documents were extracted from the National Water Authority’s repository of historical and special publications^20^, and the physical documents were obtained from the national agricultural library at the National Agrarian University La Molina. When digitized versions contained illegible soil data or map information, we consulted the physical copies. The ZEE were primarily extracted from the GeoServer of the Ministry of Environment^21^. We identified 43 ZEE reports, of which 18 provided valid soil data. For reports unavailable in this repository, official requests were submitted to the respective regional, provincial, or district governments under the Transparency and Public Information Access Law^22^. Research studies were primarily sourced from the Peruvian national registry of research works – RENATI^23^, with additional scientific articles identified through systematic searches in Latindex, Scopus, and Web of Science databases or via recommendations from local researchers. This process yielded a total of 103 identified documents.

The soil data were structured in a tabular format, following the procedures used for the WoSIS database^24^. Soil survey data typically describe soil profiles (pedons) that are composed of one or more soil samples taken at various depths. The data were organized into three independents but interrelated UTF-8 encoded CSV files: a dictionary file describing the meaning of each column header; a profile file detailing the main attributes of the entire soil profile (coordinates, taxonomy); and a layer file containing morphological data or physical and chemical analyses for each depth.

**Fig. 1:**
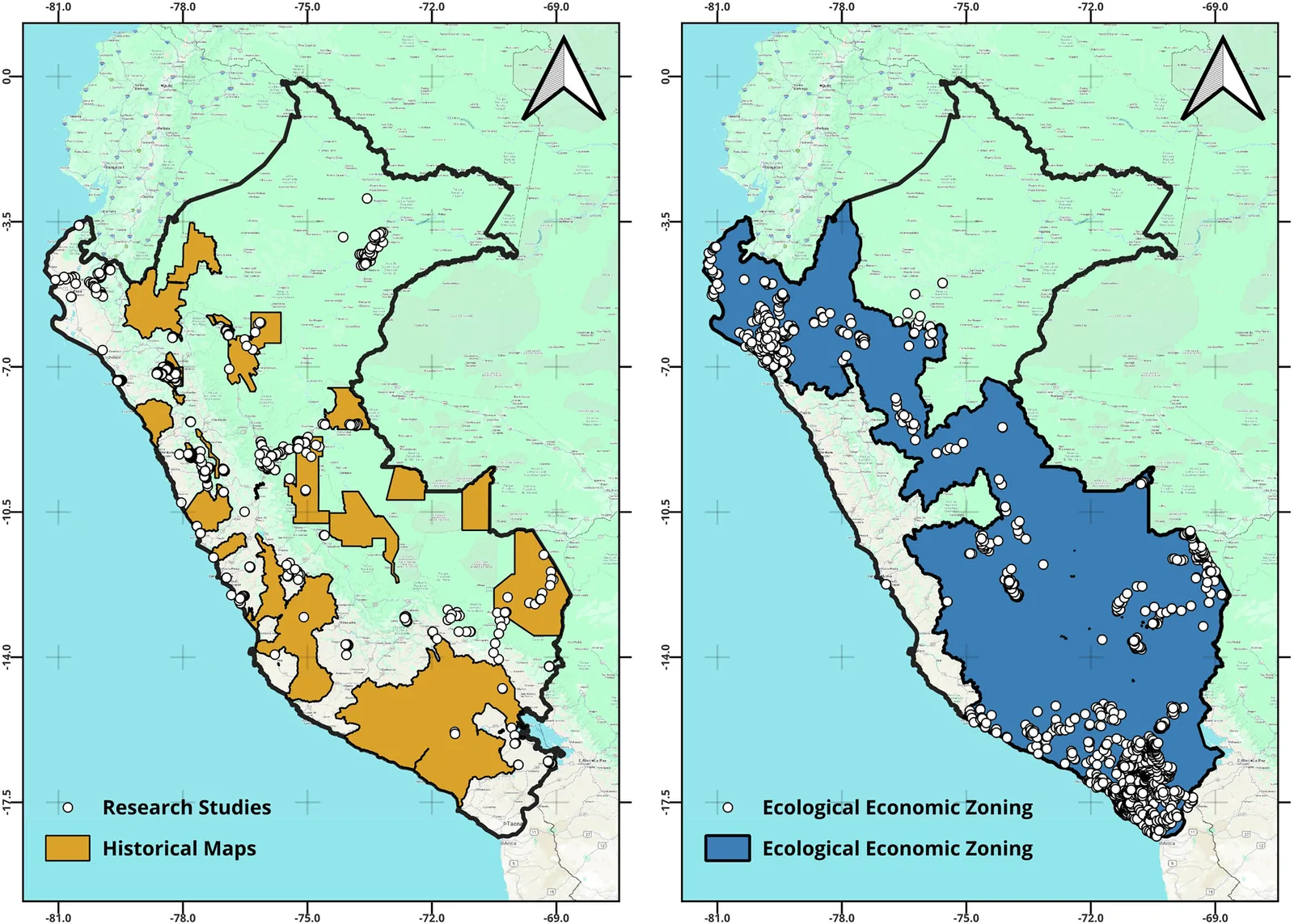
Spatial extent of maps from historical documents (left) and governmental documents (right), including the distribution of georeferenced samples from research studies and governmental data.

The maps collection was restricted to HD and ZEE maps. RES documents were not included, as most of them contained georeferenced locations that did not require mapping. In total, 83 HD and 17 ZEE maps were digitized, with the former covering 25.17% of Peru’s territory and the latter covering 59.56% (Fig. 1). These maps collectively allowed the digitization of 8793 soil profiles, including samples with only a single depth. Of these, 39% were georeferenced, with 1594 profiles originating from ZEE documents and 1856 from RES documents.

### Laboratory methods for soil analysis

Soil characterization analyses have been routinely performed on soil samples since the establishment of the Soil Laboratory at the National Agrarian University La Molina in 1958. The standard routine analyses include: pH, electrical conductivity, extractable phosphorus, carbonates, extractable acidity, organic matter, soil texture (sand, silt, clay), cation exchange capacity, extractable cations (calcium, magnesium, potassium, sodium), and extractable acidity (aluminium and hydrogen). Additional analyses may also include nitrogen, gypsum and sand fractionation.

**Fig. 2:**
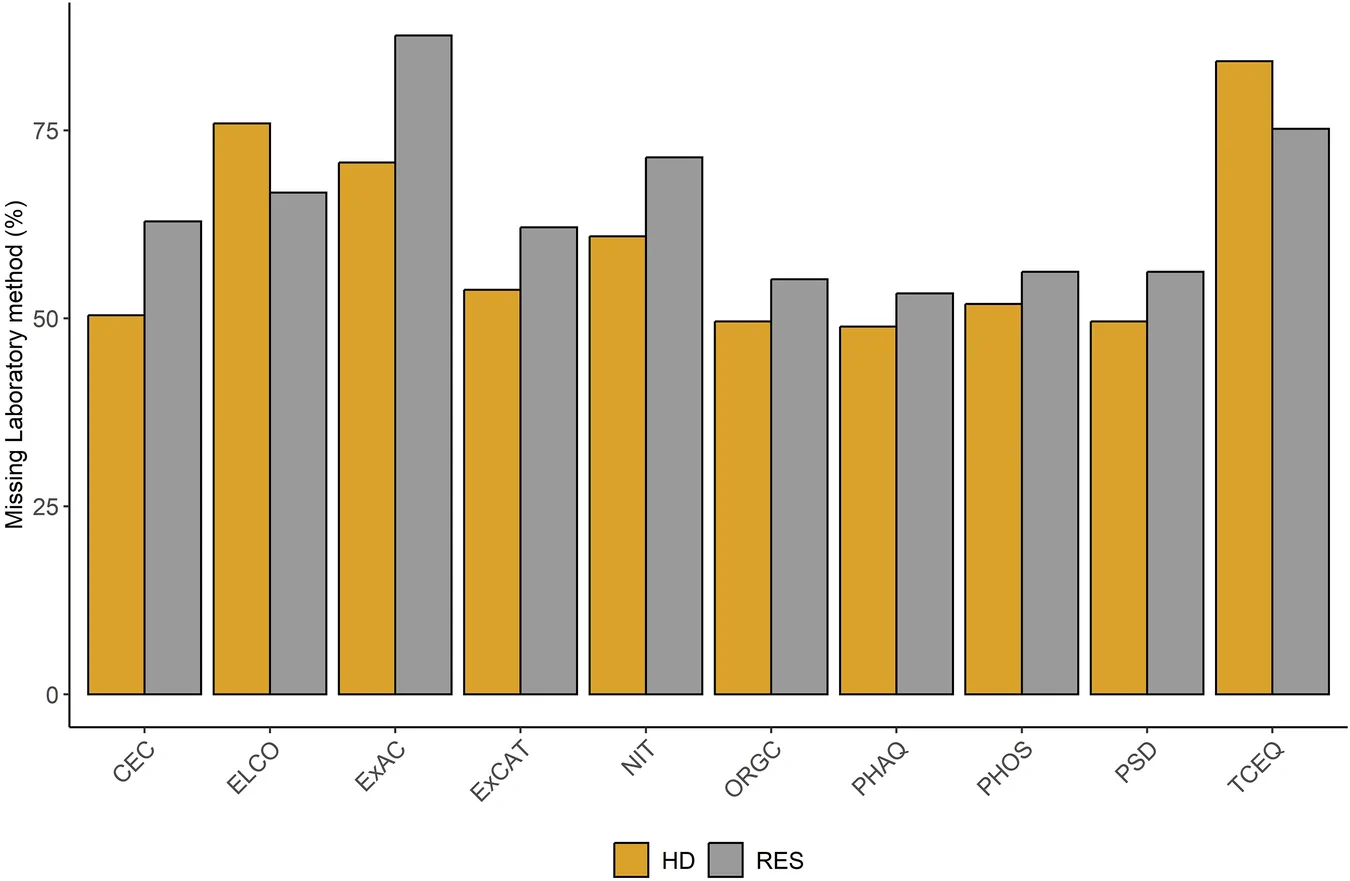
Percentage of historical (HD) and research (RES) documents lacking reported laboratory methods for cation exchange capacity (CEC), electrical conductivity (ELCO), extractable acidity (ExAC), extractable cations (ExCAT), nitrogen (NIT), organic carbon (ORGC), pH (PHAQ), extractable phosphorus (PHOS), particle size density (PSD) and carbonates (TCEQ).

The laboratory methods used followed the procedures described by Bazan^25^, which are mostly based on the different chapters described in Norman^26^. Some documents within the collection may report results for the same variable derived from different analytical methodologies. A significant proportion of the source documents (71%) lack a detailed description of the laboratory methods employed (Fig. 2), whereas all documents originating from ZEE contained a complete methodological description. Given that the Soil Laboratory of the National Agrarian University operates under national mandates, and the dataset primarily comprises routine analyses, it was a necessary and justified assumption that the unreported methods adhered to the national standard protocol. The database includes two files that provide detailed information on the laboratory methods used for each soil variable in data collection.

### Data processing

The initial step of data processing involved the direct digitization of both non-spatial soil profile information (layer data) and spatial map data from the source documents. The raw soil layer data were then manually reviewed for quality control to ensure data integrity and consistency. After this thorough review and correction process, a final, corrected layer dataset was generated. This final version, which ensures the integrity and consistency of the soil profile data, is included in the data collection.

The maps attached to each soil survey were processed digitally using QGIS 3.34.15. First, the HD maps were georeferenced to either a Geographic or Projected Coordinate System, based on the coordinate values reported on the map. The PSAD56 datum, which was the official geodetic system used in cartographic studies in Peru until the 1990s, was adopted for this process. The raster maps were saved in GeoTIFF format and then re-projected to the WGS84 datum. They were then overlaid on a Google Satellite base map to digitize the polygons using the QGIS digitize tool. As a final step, the maps underwent a quality control review to verify that their boundaries aligned with the political or geographical location specified in each study. Maps from the ZEE were downloaded directly in their vector format and with the UTM projected coordinate system, corresponding to their respective zones. All vector maps were then re-projected to a Geographic Coordinate System (EPSG: 4326) and stored in GeoPackage format.

The attribute information for each map was reorganized to a standard structure containing only three columns: label (the soil map unit symbol), Dominant Soil (the dominant soil taxa or the name of the mapping unit), and SubDominant Soil (the secondary soil taxa or NA value if not specified). This standardization allows all maps to be integrated into a single file and enables consistent operations across the entire collection.

### Data harmonization

A data harmonization process was implemented to ensure the comparability of variables analyzed with different methodologies across the compiled studies. This involved transforming values of specific variables into a single, standardized unit for consistent analysis. The variables identified as requiring this harmonization were electrical conductivity, cation exchange capacity, extractable phosphorus, pH and bulk density.

For electrical conductivity, values measured in different soil-to-water ratios were transformed to saturated paste. When 1:1 soil-to-water ratio, sand, and carbonate data were available, a pedotransfer function from Mestanza *et al*.^27^ was used. In cases where only 1:1, 1:2.5, or 1:5 soil-to-water ratio values were provided, transformations were performed using the equations proposed by Sonmez *et al*.^28^. Similarly, for cation exchange capacity, values from the ammonium acetate buffered method at pH 4.8 were transformed to their pH 7 equivalent using the equation proposed by Purnamasari *et al*.^29^. The sodium acetate method at pH 8.2 was considered equivalent to the effective cation exchange capacity^30^. For available phosphorus, values obtained with the Bray method were transformed to the Olsen method using the equation by Wolf & Baker^31^, while values from the Egner and Morgan methods were excluded from the analysis (assigned as NA). Bulk density measurements were preserved in their original form, while missing values were estimated using pedotransfer functions from Mestanza *et al*.^32^, provided the necessary predictor variables were present. Finally, no mathematical harmonization was applied to pH; instead, measurements from different methods (e.g., PHAQ, PHKC, and PHNF) were retained in separate columns due to their distinct applications and interpretations.

The code used for these harmonization processes is stored in the allpaDB_CORRECTED_harmonization.R file, and the final harmonized dataset is available as allpaDB_layer_HARMONIZED_v1.csv.

## Data Records

The compiled soil dataset is publicly available on Zenodo^33^ at https://doi.org/10.5281/zenodo.20077332. To facilitate direct access and interoperability, the dataset is presented in a flat-file structure, with files categorized by their specific functions. Technical and analytical metadata are provided in allpaDB_LaboratoryMethods_v1.csv and allpaDB_LaboratoryDescription.pdf, the former records the laboratory analyses for each document, while the latter describes the specific laboratory methodologies used. Geospatial data, including vectorized maps from historical and governmental sources, are available as geopackage files (HD_GIS_vector_p.zip and ZEE_GIS_vector_p.zip). The geospatial data also includes a collection of digitized and georeferenced historical maps, which are provided as raster layers within the compressed file HD_GIS_raster.zip. These files allow for the spatial overlay and analysis of legacy soil information with modern geographic datasets. The processing and harmonization workflows are documented in two R scripts: allpaDB_RAW_processing.R and allpaDB_CORRECTED_harmonization.R. Soil data is provided at different processing stages: raw data (allpaDB_layer_RAW_v1.csv), manually corrected data (allpaDB_layer_CORRECTED_v1.csv), profile descriptions (allpaDB_profile_v2.csv), and the final harmonized dataset (allpaDB_layer_HARMONIZED_v1.csv), supported by a variable dictionary (allpaDB_dictionary_v1.csv). Finally, the list of bibliographic references was integrated directly into allpaDB_profile_v2.csv as a new column named “Reference”, linked exclusively to the soil profiles that had available geographic coordinates. All tabular data files (.csv) are provided with UTF-8 encoding to support special characters.

## Technical Validation

### Quality control of raw data

The raw data underwent a thorough quality control process to identify and correct typographical errors and structural inconsistencies. This manual review and correction procedure followed these steps:

- **Variable Structure and Data Type Validation**. We used the str() function in R to check for correct variable structures. This allowed us to identify and correct common data type errors, such as the use of a comma (,) as a decimal separator, double periods (..) instead of a single decimal point, or cells that were incorrectly recognized as a different variable type.
- **Numeric range and Consistency Checks**. All data values were reviewed for inconsistencies with their expected range. This included correcting depths greater than 200 cm and ensuring that all percentage variables were between 0% and 100%. For instance, we verified that percentage values were not negative or above 100%.
- **Addressing Duplicate Profile Identifiers**. The aqp library^34^ and its checkHzDepthLogic function were used to identify soil profiles with duplicate names (profile_code). This common issue arises when pedologists in different regions assign the same common name to distinct soils. To resolve this, we generated unique profile identifiers by concatenating the source document code (doc_code) with the original profile name (profile_code). This procedure effectively disambiguated profiles sharing identical names across different legacy studies, ensuring each record was uniquely identified within the dataset.
- **Correcting Depth Data Inconsistencies**. We addressed inconsistencies in depth data, such as missing values, overlapping depths or other logical errors, by cross-referencing with the original documents. These were corrected by cross-referencing with the original documents. For studies that indicated a horizon continued without specifying a bottom depth, we assigned the nearest standard depth value from a predefined list (15, 30, 50, 75, 100, 150, or 200 cm).
- **Handling Special Characters**. To prevent issues with the recognition of special characters used in Spanish, the original soil names were stored in a variable named “profile_code_0”. A new variable, “profile_code”, was then created where these special characters were transformed into a suitable format for English-language systems. For example, accents (tildes) were removed, and characters with diacritics were replaced with their non-diacritical counterparts. This procedure was carried out using the stringi library^35^ in R. In addition to this procedure, all final CSV files were exported using UTF-8 encoding to ensure the proper reading and preservation of the original metadata and Spanish characters in the remaining variables.

A total of 7564 soil profiles were identified in the raw data, of which 1554 exhibited at least one type of depth inconsistency. These included depth logic (bottom depth shallower than top depth), same depth (equal top and bottom depth), missing depth (missing top or bottom depth), and overlap or gap depth (gap or overlap between adjacent horizons). Notably, these categories were not mutually exclusive; a single soil profile could exhibit multiple types of inconsistencies simultaneously. Following the inconsistency correction process, the dataset increased to 8793 profiles. This increment occurred because several records initially flagged as same depth errors were identified as distinct soil profiles sharing identical names. In the absence of a national soil series standardization in Peru, it is a common practice for pedologists in different regions to assign non-unique identifiers based on local landmarks, such as “montaña” or “cementerio”, leading to name collisions across independent projects. Table 1 provides a detailed description of the identified inconsistencies and the subsequent corrections performed. The correction process achieved a 100% success rate for both depth logic and same depth errors, and a 97.3% rate for overlap or gap depth discrepancies. Conversely, the remaining 865 profiles with missing depth errors could not be corrected, as the original legacy studies did not report the required sampling depths.

**Table 1:** Summary of identified and corrected inconsistencies: transition from the raw dataset to the corrected data.

Category	Raw Data	Corrected Data	Technical Outcome
Total Soil Profiles	7564	8793	+1229* (Disambiguated)
Profiles with Inconsistencies	1554	885	43% reduction
Depth Logic	156	0	100% corrected
Same Depth	7	0	100% corrected
Overlap or Gap	736	20	97.3% corrected
Missing Depth	867	865	Uncorrectable

*Note:* *The increase in total profiles reflects the disambiguation of records sharing identical names, which were correctly identified as distinct individuals during the validation process.

### Quality control of Maps

All digitized maps were reprojected to the Geographic Coordinate System with the WGS84 datum (EPSG: 4326). The QuickMapServices plugin was then used to overlay the maps on a Google Satellite base map, which allowed for visual verification that each map was correctly located within its corresponding territory. Additionally, the official departmental boundary data from the National Geographic Institute^36^ was used to cross-verify that the map boundaries aligned with the official administrative limits of Peru.

### Data consistency

After obtaining the harmonized dataset, its consistency was reviewed based on several theoretical aspects to ensure the dataset’s reliability. At sites with soil pH below 7.0, soil samples were generally expected to not contain carbonates^37^. For samples that met this condition but had no reported carbonate value, a value of zero was assigned. Given the theoretical basis of gibbsite hydrolysis, extractable aluminum generally occurs in soils with a pH value less than 5^38^. Samples with a pH greater than 5 and no reported extractable acidity value were assigned a value of zero. In cases where only a value for extractable aluminum was provided, it was directly assigned as the extractable acidity. Finally, for instances where the effective cation exchange capacity was not reported, it was determined by calculating the sum of basic and acidic cations.

## Data Availability

The compiled soil dataset described in this Data Descriptor is publicly available on Zenodo at the following URL: https://doi.org/10.5281/zenodo.20077332.

## Code availability

The code used to process and generate the soil dataset is publicly available on Zenodo at https://doi.org/10.5281/zenodo.20077332. This includes the R scripts for processing the raw data (allpaDB_RAW_processing.R) and the data harmonization procedure (allpaDB_CORRECTED_harmonization.R). All analysis and processing were conducted using R version 4.4.2.
